# Sleep Hygiene Improves Aerobic and Anaerobic Performance Independent of Cortisol Mediation in Female Collegiate Soccer Players

**DOI:** 10.3390/jfmk11020187

**Published:** 2026-05-07

**Authors:** Elric Pretorius, Mark Kramer, Adele Broodryk

**Affiliations:** Physical Activity, Sport and Recreation Research Focus Area (PhASRec), Faculty of Health Sciences, North-West University, Potchefstroom 2531, South Africa; elricpretorius13@gmail.com (E.P.); mark.kramer@nwu.ac.za (M.K.)

**Keywords:** sleep, cortisol, female, soccer, aerobic, anaerobic, sleep hygiene

## Abstract

**Background**: Sleep hygiene protocols (SHPs) have been shown to improve sleep and stress regulation; however, the role of cortisol in shaping downstream physiological and performance adaptations remains unclear. This study primarily examined the effects of a short-term SHP on sleep duration and salivary cortisol responses across resting, pre-exercise, and post-exercise states in female collegiate soccer players and, secondarily, whether cortisol statistically mediated selected aerobic and anaerobic performance outcomes. **Methods**: Fourteen players (22.1 ± 3.3 y; 157.8 ± 6.0 cm; 53.5 ± 3.9 kg) completed a randomised, counterbalanced crossover study comparing habitual sleep (no sleep hygiene protocol; nSHP) with a comprehensive SHP incorporating environmental, behavioural, and educational strategies. Salivary cortisol was sampled one hour post-waking and 30 min pre- and 15 min post-exercise during standardised testing sessions. Performance outcomes included vertical jump, sprint performance (40 m and repeated sprints [RAST]), and the Yo-Yo Intermittent Recovery Test Level 1. Linear mixed-effects models assessed cortisol responses, and mediation analyses explored cortisol–performance relationships. **Results**: After SHP, perceived (7.87 h vs. 6.5 h; *p* = 0.002, ESg = 1.0) and calculated sleep duration (8.5 h vs. 6.9 h; *p* = 0.004, ESg = 0.95) increased significantly. Cortisol was markedly lower following SHP at selected timepoints, including before RAST (−43.05%, *p* = 0.006, ESg = 0.84), with additional timepoint-specific, condition-dependent differences post-anaerobic and post-aerobic exercise (Δ = 7.37 and 5.98 nmol·L^−1^, respectively; *p* < 0.001). Vertical jump height demonstrated significant total (9.92 cm, *p* = 0.002) and direct effects (7.72 cm, *p* = 0.034), and peak repeated-sprint performance showed a significant direct effect (*p* = 0.026). Cortisol did not significantly mediate any performance outcomes (ACME *p* > 0.05). **Conclusions**: Short-term sleep hygiene is associated with increased sleep duration, timepoint-specific modulation of cortisol responses, and selected anaerobic performance benefits; however, these effects were not explained by measured cortisol responses and are unlikely to be sustained without ongoing reinforcement or support, particularly in athletic populations.

## 1. Introduction

Sleep quality and duration are fundamental to athletic recovery and performance in competitive sports [1]. Female collegiate soccer players face unique physiological and psychosocial challenges that may compromise sleep patterns, including academic demands, training loads, social pressures, and hormonal fluctuations, which collectively affect recovery markers and performance outcomes [2,3]. Despite growing interest in sleep optimisation, evidence supporting consistent benefits of sleep hygiene interventions for performance and physiological stress regulation remains mixed. While some studies suggest that targeted sleep hygiene strategies can improve sleep duration and selected performance outcomes [4,5], other investigations report minimal or task-specific effects, indicating that sleep–performance relationships are context-dependent rather than universal.

In female athletes competing in high-demand intermittent sports such as soccer, sleep restriction causes profound physiological and psychological impairments. Poor sleep has been shown to increase the salivary cortisol area under the curve (AUC) by approximately 42%, alongside reductions in lower-body power output and resistance exercise quality [6]. Elevated cortisol may contribute to delayed muscle repair, impaired glycogen restoration, and disrupted anabolic–catabolic balance [7]. In parallel, sleep loss impairs cognitive processes, including reaction time and decision-making accuracy, potentially undermining technical execution and tactical performance [8,9]. However, these responses are not uniform across studies, with performance decrements often varying by task type, timing, and assessment method.

The hypothalamic–pituitary–adrenal (HPA) axis, with cortisol as its primary effector hormone, plays a central role in regulating stress responses, circadian rhythms, and recovery in athletes [10,11]. Cortisol supports energy mobilisation and inflammatory regulation, with moderate elevations supporting performance by increasing glucose availability [7]. Conversely, excessive or prolonged cortisol secretion has catabolic effects, impairing recovery and increasing perceived fatigue [12]. Importantly, recent work shows that cortisol responses vary with stressor characteristics and temporal context, with anticipatory elevations reflecting central nervous system activation rather than metabolic demand alone [12,13]. Soccer-specific performance decrements, such as reduced high-intensity running, sprint distance, and technical precision, are frequently observed later in matches and are exacerbated by accumulated fatigue and psychological stress [14,15]. Competitive matches consistently elicit greater cortisol elevations than training sessions, with anaerobic activities producing larger hormonal responses than aerobic exercise due to their higher neuromuscular and psychological demands [12,13,16,17].

The relationship between cortisol dynamics and performance remains unclear. Some studies report associations between elevated cortisol and reduced neuromuscular performance, including lower vertical jump height after high-intensity training periods [11]. In contrast, several investigations document improvements in aerobic and anaerobic performance without concurrent changes in basal cortisol concentrations, suggesting that performance adaptations may occur independently of measurable hormonal changes [17,18,19]. Such discrepancies may stem from methodological differences, including reliance on single-timepoint hormone measures, male-dominant samples, and non-individualised intervention designs.

Cortisol exhibits a pronounced diurnal rhythm, peaking shortly after waking as part of the cortisol awakening response (CAR) and declining throughout the day [20]. This rhythm is sensitive to external stressors, including sleep restriction, late-night exercise, and psychological strain [21,22,23]. Even a single night of restricted sleep (<6 h) can elevate cortisol by approximately 45% [24], and chronic sleep disturbances and subsequent cortisol increases are linked to impaired recovery and increased injury risk [22,25,26,27]. However, most sleep-hygiene studies assess cortisol only at rest, overlooking how anticipatory and post-exercise responses diverge across anaerobic and aerobic stressors, an omission particularly relevant in female athletes with hormonally sensitive stress regulation [26].

Individualised sleep hygiene interventions, including sleep education, environmental optimisation, and behavioural strategies, have shown promise in improving sleep quality and quantity among female soccer players [26,28,29,30]. Improvements in sleep duration, latency, and countermovement jump performance have been reported after targeted interventions [31]. Acute sleep hygiene strategies have also been shown to modify salivary cortisol time courses [28] and various sleep indices [32,33,34] following late-evening training or competition. Nevertheless, it remains unclear whether cortisol plays a mechanistic role in translating sleep improvements into performance adaptations, or whether observed performance benefits arise through alternative pathways, such as enhanced neuromuscular readiness or cognitive recovery [35,36].

Critically, few studies have examined dynamic cortisol responses (waking, pre-exercise, post-exercise) alongside aerobic and anaerobic performance outcomes within the same experimental framework. Most existing research relies on single-point or basal measurements, limiting mechanistic interpretation [37]. No study to date has systematically evaluated whether changes in cortisol statistically mediate sleep-related performance adaptations in female collegiate soccer players using a within-subject crossover design. Addressing this gap is essential for clarifying the physiological pathways through which sleep optimisation influences recovery and performance.

Therefore, this study aimed to investigate the relationship between changes in salivary cortisol and changes in aerobic and anaerobic performance following a sleep hygiene protocol (SHP) in female collegiate soccer players, using a randomised, counterbalanced crossover design. We hypothesised that SHP would reduce cortisol concentrations at waking and before exercise, attenuate post-exercise cortisol responses, and improve selected anaerobic performance outcomes, reflecting improved stress regulation across basal, preparatory, and recovery states. Furthermore, we hypothesised that cortisol changes would show limited mediation of performance adaptations, indicating predominantly non-hormonal mechanisms.

## 2. Materials and Methods

### 2.1. Experimental Approach to the Problem

A randomised, counterbalanced crossover design was used, with participants completing both a sleep hygiene protocol (SHP) and a no-intervention condition (nSHP), separated by a 14-day washout. Participants were randomly assigned to the SHP → nSHP or nSHP → SHP sequence using a computer-generated schedule. Given the behavioural nature of the intervention, allocation concealment beyond the initial sequence assignment was not feasible. Data collection included validated questionnaires, physical performance assessments, and systematic observations. Ethical approval was obtained from the institutional review board (NWU-00299-21-A1), and all participants provided written informed consent in accordance with the Declaration of Helsinki.

### 2.2. Participants

Initially, 20 female players volunteered for the study. Six participants were excluded: one due to injury, one for insufficient saliva samples, two for failing to complete post-testing, and two for incomplete adherence to the sleep protocol. The final sample comprised 14 female players (22.1 ± 3.3 y, 157.8 cm ± 6.0 cm, 53.5 ± 3.9 kg) from a tertiary academic institution who volunteered for the study. The sample comprised the entire first-team squad from a tertiary academic institution, and no additional eligible participants were available for recruitment during testing. An a priori power analysis for a repeated-measures within–between interaction (α = 0.05, β = 0.20, expected correlation = 0.70) assumed a moderate effect size (f = 0.25), yielding a minimum target sample size of 14 participants.

### 2.3. Salivary Cortisol

Saliva samples were collected to assess cortisol using the passive drool test [38]. Salivary cortisol was collected at three standardised timepoints on each testing day: (1) one hour after waking in a fasted state to assess morning basal values, (2) upon arrival at the sports grounds at exactly 14:00, 30 min before physical testing (pre-RAST/YYIR1), and (3) 15 min post-exercise (post-RAST/YYIR1) to capture the acute stress response [39]. These timepoints were selected to capture cortisol dynamics across physiologically relevant states (basal, anticipatory, and post-exercise) while minimising participant burden in an applied field-based setting. Ten minutes before sampling, participants rinsed their mouths with lukewarm water, and saliva was collected via passive drooling into 2 mL vials while seated upright. Samples were immediately frozen (−80 ± 1 °C) and stored until analysis [38]. Cortisol content in 20 µL saliva samples was determined using the Salimetrics^®^ High Sensitivity Salivary Cortisol Enzyme Immunoassay Kit (Salimetrics, LLC, State College, PA, USA). Samples were then transferred to a Berthold luminometer (Bemis Company, Inc., Bad Wildbad, Germany) to calculate the average relative luminescence units, which show strong agreement with serum cortisol measurements (r = 0.8–1.0) and provide a reliable, non-invasive index of HPA-axis activity. Analytical reliability was high, with reported intra-assay and inter-assay coefficients of variation of 0.4–1.7% and 0.8–1.8%, respectively [40]. These characteristics support the validity and precision of the cortisol measurements used in the present study.

To minimise external influences on cortisol secretion, participants were instructed to remain awake and fasted for at least one hour before morning sampling [40]. The morning sample, collected one hour post-waking, was intended to provide a standardised basal reference point rather than a formal assessment of the cortisol awakening response, which typically requires multiple samples within the first 30–45 min after waking [28,40]. Post-exercise samples were collected 15 min after testing to align with the established temporal window for the peak salivary cortisol response following a stressor [26,41]. Given the pronounced diurnal rhythm of cortisol, awakening and collection times were logged and replicated across conditions and testing phases [40].

### 2.4. Sleep and Sporting Activity Questionnaire

Sleep behaviour, perceived fatigue, and stress were assessed using the Sleep and Sporting Activity Questionnaire (SSAQ) [42]. Sleep quality was rated on a 5-point Likert scale, with “1” indicating highly restorative sleep and “5” indicating poor rest. The SSAQ has been validated against actigraphy, demonstrating excellent reliability for time in bed (ICC = 0.93–0.95) and total sleep time (ICC = 0.90–0.92) [34]. Sleep latency was defined as the time to fall asleep; perceived sleep duration was the subjective time slept; and time in bed was the total time spent in bed, whether asleep or awake. Actual sleep time was calculated using the following formula:

Total Sleep Time = (Time in bed) − (Sleep onset latency) − (Time awake after sleep onset).

### 2.5. Physical Performance Assessments

#### 2.5.1. Forty-Metre Sprint Test

Sprint performance was measured on a soccer field using electronic timing gates (SmartSpeed, Fusion Sport, Brisbane, QLD, Australia) positioned at 0, 5, 10, 20, and 40 m [43]. Linear sprint tests show high reliability (ICC > 0.75; CV < 3%), supporting their consistent use in soccer performance assessment [43]. Athletes wore soccer boots for ecological validity and completed two sprint trials, separated by a 2 min rest. Split times were recorded at 5, 10, and 20 m, and total sprint time was recorded at 40 m. All times were measured to the nearest 0.01 s, and the fastest trial was retained for analysis.

#### 2.5.2. Vertical Jump Test

Explosive lower-body power was assessed using a vertical jump test (VJT) with a Vertec device (Sports Imports, Inc., Columbus, OH, USA). Vertical jump assessments demonstrate excellent reliability across measurement approaches, with reported correlation coefficients typically exceeding r > 0.97 [44]. Additionally, neuromuscular characteristics relative to each participant’s bodyweight were quantified using a Tendo™ Power Output Unit (Tendo Sports Machines, Trencin, Slovakia) [45]. The unit’s linear position transducer, attached at the waist, measured vertical displacement and jump duration to calculate jump velocity and power output. During each attempt, athletes performed a vertical jump with an arm swing. Jump height was calculated as the difference between standing reach and the highest vane touched. Each participant performed two jumps, separated by a 2 min rest interval, and the best result was analysed. Peak and mean power (W) and velocity (m·s^−1^) were also derived for each jump. Test–retest reliability for the Tendo unit has consistently shown r > 0.90 [45].

#### 2.5.3. Yo-Yo Intermittent Recovery Test Level 1 (YYIR-1)

Intermittent endurance was assessed using the YYIR1, a valid and reliable field test that correlates with the amount of high-intensity running performed during a match (r = 0.81) and with VO2max (r = 0.7) [46]. The test was conducted on a standard soccer field with players wearing soccer boots. It involved 20 m shuttle runs paced by audio signals, with 10 s of active recovery. Recorded parameters included total distance, number of shuttles, and maximum heart rate (Polar H10 heart rate monitor and belt, Polar Electro, Kempele, Finland).

#### 2.5.4. Repeated Anaerobic Sprint Test (RAST)

High-intensity, short-duration performance was assessed using the validated RAST protocol [47], comprising six 40 m shuttle sprints with 20 s passive recovery. The RAST is a commonly used and validated repeated-sprint protocol for team-sport athletes, demonstrating strong ecological validity and high reliability for time-based performance outcomes (ICC = 0.88–0.99; CV ≤ 5%) [43,47]. A baseline sprint was recorded using a photocell system (SmartSpeed, Fusion Sport, Brisbane, QLD, Australia), followed by a 5 min recovery. If the first sprint in the RAST exceeded the baseline by >2.5%, the test was repeated after recovery. Performance metrics included the best and mean repetition times, percentage decrements across the six repetitions, and maximum heart rate (measured with a Polar H10 heart rate monitor and belt, Polar Electro, Kempele, Finland).

### 2.6. Sleep Hygiene Protocol (SHP)

During the non-SHP condition, participants maintained their habitual sleep routines, self-regulated electronic device use, pre-bed lighting (±60 ± 12 W), and bedtime patterns. The SHP condition was implemented in participants’ home environments over 14 consecutive nights before SHP testing. Participants were instructed to adhere to at least 10 of the 18 sleep hygiene recommendations (Table 1) each day. Adherence to each component was recorded daily in a structured sleep diary, allowing calculation of both a binary compliance indicator (meeting the ≥10-component criterion) and a continuous measure of adherence (number of components achieved per day), thereby capturing between- and within-participant variability in sleep hygiene engagement.

Participants were dual-career athletes with diverse living and academic contexts, including shared residential environments, variable commuting demands, and fluctuating academic workloads. Accordingly, the sleep hygiene protocol was designed to allow context-specific customisation within a structured framework, enabling athletes to implement strategies most compatible with their environments while ensuring engagement across multiple core sleep-hygiene domains.

### 2.7. Procedures

**Figure 1:**
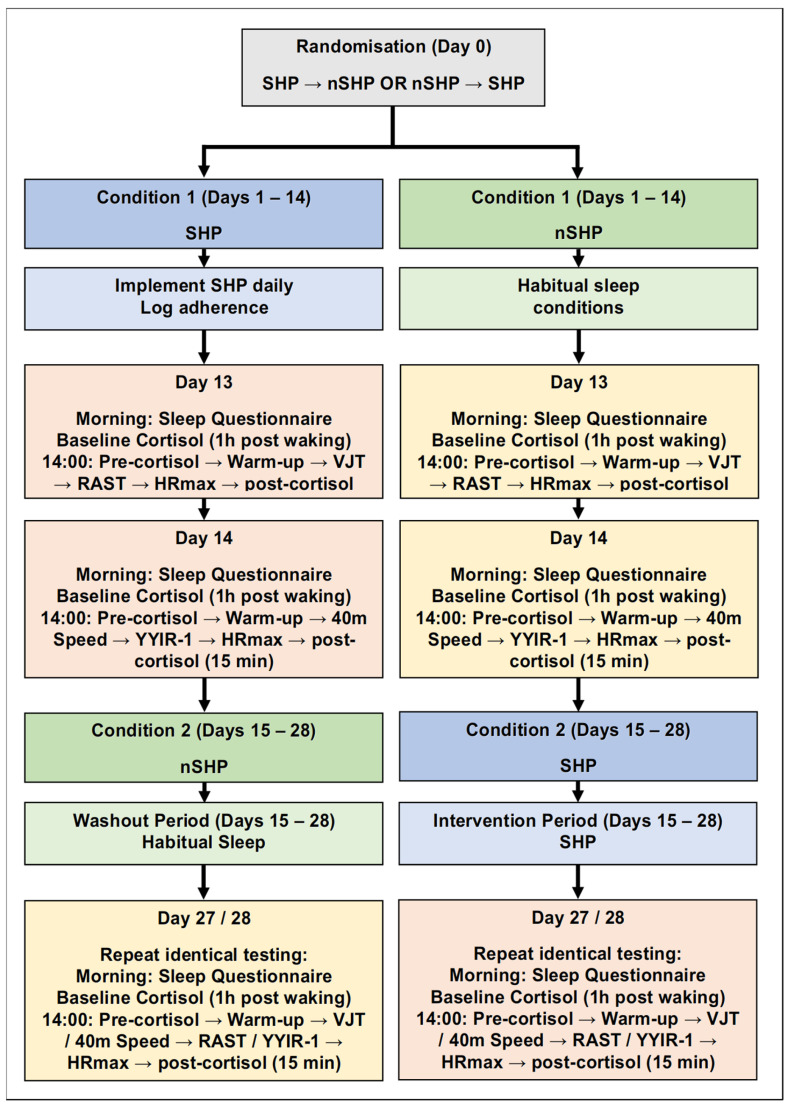
Overview of the 14-Day Randomised Crossover Protocol Comparing SHP and nSHP Conditions. Note: HRmax = Heart Rate maximum; RAST = Repeated Anaerobic Sprint Test; SHP—Sleep Hygiene Protocol; nSHP—No Sleep Hygiene Protocol; YYIR-1 = Yo-Yo Intermittent Recovery test Level 1; VJT = Vertical Jump Test.

The study used a randomised, counterbalanced crossover design comprising a 14-day sleep hygiene protocol (SHP) and a 14-day no-sleep hygiene phase (nSHP), separated by a 14-day washout period (Figure 1). During the familiarisation session, participants were randomly assigned to complete either the SHP followed by nSHP or the reverse order. At the start of the SHP phase, athletes received a structured instructional session and written guidelines outlining 18 evidence-based sleep hygiene recommendations.

They were instructed to implement at least 10 of these strategies consistently each day for 14 consecutive days and to log daily adherence. During the nSHP condition, participants maintained their habitual sleep behaviours and received no behavioural instruction.

Physical-performance testing and salivary cortisol sampling were conducted during the final two days of each 14-day condition to ensure adequate exposure to the relevant sleep condition before assessment. On the first testing day (Day 13), participants completed a sleep questionnaire upon waking and provided a saliva sample one hour later, which served as the condition-specific baseline. At 14:00, they reported to the soccer field for a second saliva sample before exercise, followed by a standardised 15 min dynamic warm-up, then the vertical jump and repeated-sprint ability test (RAST). A third saliva sample was collected 15 min after completion of the exercise protocol. On the second testing day (Day 14), participants completed a standardised warm-up, followed by the 40 m sprint and the Yo-Yo Intermittent Recovery Test Level 1 (YYIR-1), with a post-exercise saliva sample collected 15 min thereafter. The same testing procedures were repeated identically after completion of the alternate condition.

Participants abstained from caffeine, alcohol, and vigorous physical activity for at least 48 h before each testing block and fasted for at least one hour before saliva sampling. Dietary intake, training load, and lifestyle behaviours were standardised and kept consistent across conditions. All testing was conducted during the pre-season period at the start of the academic term to minimise academic-related stress. To reduce environmental confounding, testing sessions were scheduled on comparable weekdays in line with the team’s maintenance training schedule and usual training times. Minor environmental factors (e.g., light wind conditions) were accommodated through a standardised test setup and orientation.

### 2.8. Statistical Analyses

All analyses were conducted using R (RStudio, version 2023.06.1, Posit Software, Boston, MA, USA) and IBM SPSS Statistics for Windows (version 31, IBM Corp., Armonk, NY, USA). Descriptive statistics are reported as mean ± standard deviation unless otherwise stated. Perceived and objectively measured sleep duration were predefined primary outcomes. Sleep latency and sleep quality scores were analysed as secondary outcomes and interpreted conservatively; therefore, no adjustment for multiple comparisons was applied to primary sleep outcomes. Standardised effect sizes are reported as Hedge’s g and interpreted as trivial (<0.20), small (0.20–0.50), medium (0.50–0.80), large (0.80–1.20), very large (1.20–2.0), and huge (>2.0) [48].

Linear mixed-effects models (LMMs) were used to analyse cortisol and performance outcomes, accounting for repeated measures and inter-individual variability. Participants were included as random intercepts in all models. Fixed effects included Condition (sleep hygiene protocol [SHP] vs. no sleep hygiene protocol [nSHP]), Time (baseline, pre-exercise, post-exercise), and the Condition × Time interaction to formally test whether changes in cortisol or performance differed as a function of the intervention over time. Menstrual cycle phase (follicular, luteal) was included as a fixed covariate in all hormonal and performance models, given its known influence on stress-hormone regulation. Random slope terms were omitted to avoid over-parameterisation, given the modest sample size. Time-specific contrasts were extracted from significant interaction effects and adjusted using the Holm correction to control the family-wise error rate.

Mediation analyses were conducted to assess whether cortisol statistically mediated the effect of the sleep hygiene intervention on performance outcomes. Total effects were decomposed into an average direct effect (ADE) and an average causal mediation effect (ACME). The significance of indirect effects was estimated using the Sobel method; however, this approach may be underpowered in small samples and assumes normality of the indirect effect distribution. Accordingly, results should be interpreted with caution. Given the crossover design, modest sample size, and the absence of specific mechanistic measures, the mediation analyses were treated as exploratory and hypothesis-generating rather than tests of causal inference. Model assumptions, including residual normality and homoscedasticity, were evaluated using residual plots and deemed adequately satisfied; mediation results were interpreted cautiously.

## 3. Results

Following the SHP, participants reported significantly longer perceived (SHP = 7.87 h, nSHP = 6.5 h, *p* = 0.002, ESg = 1.0, CI: 1.65–0.35) and measured (SHP = 8.5 h, nSHP = 6.9 h, *p* = 0.004, ESg = 0.95, CI: 0.31–1.57) sleep times. No significant differences were observed in sleep latency (SHP = 19.46 min, nSHP = 25.7 min, *p* = 0.22, ESg = 0.35, CI: −0.22–0.91) or sleep scores (SHP = 2.63, nSHP = 2.88, *p* = 0.49, ESg = 0.33, CI: −0.50–0.85).

Table 2 summarises percentage changes in salivary cortisol across key timepoint comparisons under two experimental conditions: non-sleep hygiene (nSHP) and sleep hygiene (SHP). Menstrual cycle phase was not a significant predictor of salivary cortisol concentrations in the mixed-effects models (all *p* > 0.05) and was therefore retained as a covariate without further stratification.

Across conditions, salivary cortisol responses were consistently lower following the SHP condition across testing timepoints. Anticipatory cortisol concentrations before anaerobic exercise (RAST) decreased by 43.05% (*p* = 0.006, ESg = 0.84, CI: 0.24–1.40), while pre-aerobic (YYIR-1) cortisol levels showed a 25% reduction, corresponding to a medium effect size (*p* = 0.060, ESg = 0.55, CI: 0.04–0.83). In addition, post-aerobic cortisol concentrations were markedly lower following SHP, with a 55.78% reduction relative to habitual sleep (*p* = 0.004, ESg = 0.90, CI: 0.28–1.50). Within-condition comparisons revealed significant changes only in the nSHP group, with cortisol decreasing by 39.23% from pre- to post-anaerobic testing (*p* = 0.01, ESg = 0.68, CI: 0.16–1.18) and increasing by 58.62% from pre- to post-aerobic testing (*p* = 0.035, ESg = 0.57, CI: 0.19–0.85). All within-condition contrasts were derived from the linear mixed-effects models, and the corresponding *p*-values reflect Holm-adjusted post hoc comparisons extracted from these models.

**Figure 2:**
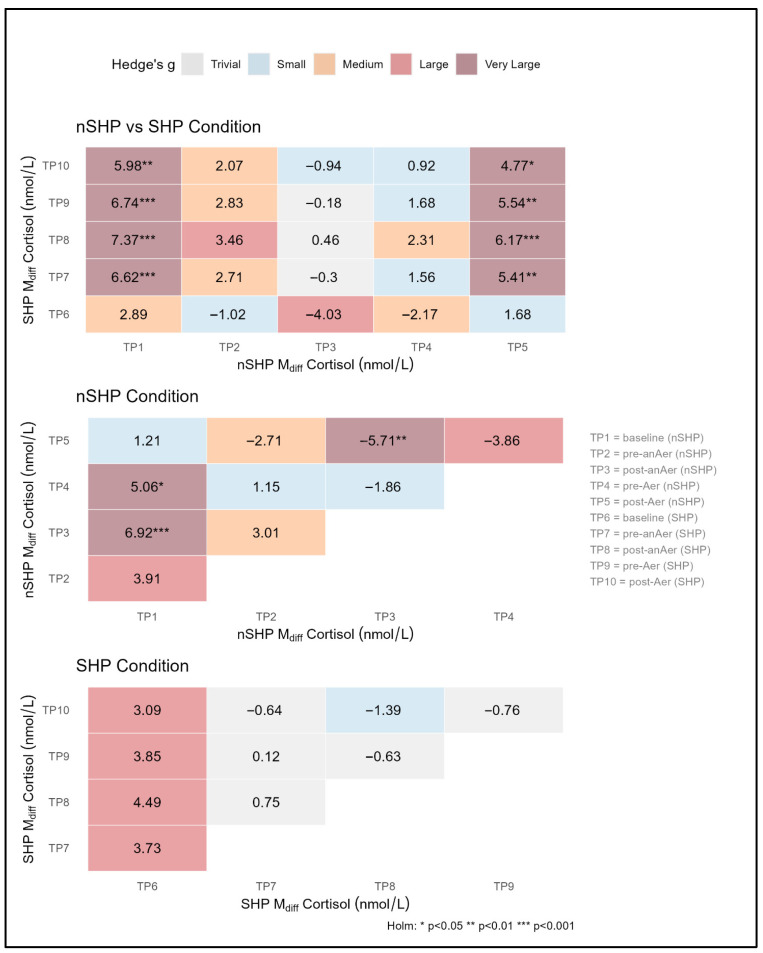
Model-based comparison of cortisol responses across conditions and timepoints. Note: Tiles show the mean differences in cortisol values across timepoints and conditions and are coloured based on the magnitude of the standardised effect size (Hedge’s g). Asterisks denote Holm-adjusted statistical significance (* *p* < 0.05, ** *p* < 0.01, *** *p* < 0.001). TP = timepoint; SHP = sleep hygiene protocol; nSHP = no sleep hygiene protocol.

Figure 2 presents model-derived contrast estimates of salivary cortisol across timepoints under nSHP and SHP conditions. Between-condition analyses revealed significant differences favouring SHP at multiple post-exercise timepoints. Cortisol concentrations were significantly lower following SHP than nSHP after RAST (Δ = 7.37 nmol·L^−1^, *p* < 0.001) and at pre- (Δ = 6.74 nmol·L^−1^, *p* < 0.001) and post-YYIR1 (Δ = 5.98 nmol·L^−1^, *p* < 0.001), as indicated by significant positive contrast estimates in the between-condition heatmap.

Between-condition analysis revealed significant positive contrasts following the SHP condition, with the largest differences observed post-anaerobic (Δ = 7.37 nmol·L^−1^, *p* < 0.001) and TP_9_ (Δ = 6.74 nmol·L^−1^, *p* < 0.001). Post-aerobic results also showed a significant difference (Δ = 5.98 nmol·L^−1^, *p* < 0.001). Earlier timepoints (nSHP) exhibited smaller, mostly non-significant contrasts, with post-anaerobic values showing a negative estimate (Δ = −0.94 nmol·L^−1^). Within SHP, contrasts between consecutive timepoints ranged from −1.39 to +4.49 nmol·L^−1^, with TP_6_–TP_8_ showing the highest positive estimates. Within nSHP, the range of contrasts was narrower, with values between −1.39 and +0.75 nmol·L^−1^. At baseline (TP_1_), cortisol concentrations were higher in nSHP than in SHP.

**Figure 3:**
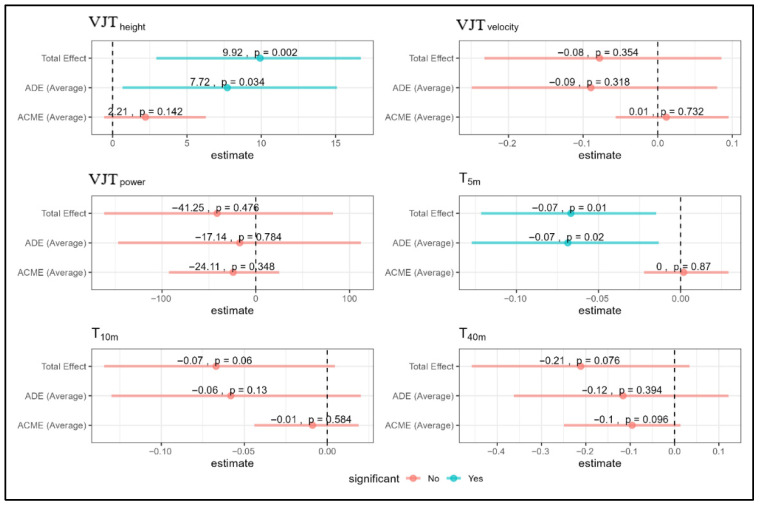
Mediation analysis of anaerobic metrics showing Total, Direct (ADE), and Mediation (ACME) effects for VJT and sprint measures. Note: blue = significant; red = non-significant; VJT = Vertical Jump Test; T = Time.

The mediation analysis of anaerobic performance (Figure 3) showed a significant total effect on VJT height (9.92 cm, *p* = 0.002) and a significant direct effect (7.72 cm, *p* = 0.034). The mediation effect was not significant (ACME = 2.21 cm, *p* = 0.142). VJT velocity showed no significant total, direct, or mediated effects. Sprint performance measures exhibited non-significant total effects, with T5 m (−0.07 s, *p* = 0.06) and T40 m (−0.21 s, *p* = 0.076) approaching significance; neither ADE nor ACME reached significance for any sprint metric. These mediation analyses were exploratory, and no evidence of statistically significant indirect effects of cortisol on performance outcomes was detected under the current model.

**Figure 4:**
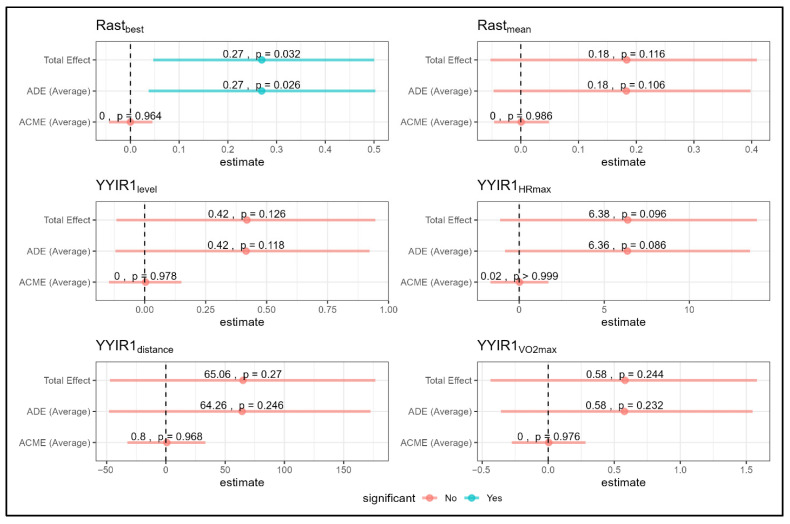
Mediation analysis of aerobic metrics showing Total, Direct (ADE), and Mediation (ACME) effects for RAST and YYIR1 variables. Note: blue = significant; red = non-significant; RAST = Repeated Anaerobic Sprint Test; YYIR1 = Yo-Yo Intermittent Recovery test 1.

The mediation analysis, as shown in Figure 4, indicated that the sleep hygiene intervention had a direct effect on peak sprint performance (RAST-best), with significant improvements observed (total effect *p* = 0.032, ADE *p* = 0.026). No mediation pathways were detected, as ACME values were consistently non-significant across all outcomes. For the remaining RAST and Yo-Yo IR1 metrics, neither direct nor mediated effects reached statistical significance.

## 4. Discussion

The primary aim of this study was to examine whether adherence to a structured sleep hygiene protocol (SHP) influenced sleep behaviour, physiological stress markers, and sport-specific performance outcomes in collegiate female soccer players, and whether cortisol mediated any observed performance effects. The main findings indicate that SHP significantly improved both subjective and objective sleep duration, was associated with timepoint-specific changes in salivary cortisol, and coincided with improvements in selected measures of explosive and sprint performance. However, these performance changes should be interpreted with caution, given potential learning or familiarisation effects associated with repeated testing, as improvements may have occurred independently of the intervention despite the crossover design and standardised procedures. Importantly, mediation analyses did not support cortisol as an intermediary mechanism, suggesting that performance changes occurred through direct, non-endocrine pathways. Collectively, these findings suggest that sleep hygiene may contribute to performance-relevant outcomes and timepoint-specific modulation of physiological stress responses, while acknowledging that the relative influence of intervention effects and testing familiarisation cannot be fully disentangled within the present study design [34,49,50].

The most robust and unambiguous effect of the sleep hygiene protocol was observed in sleep behaviour. Adherence to SHP resulted in significant increases in both perceived and objectively measured sleep duration, indicating improved sleep opportunity rather than changes in sleep quality. No significant changes were observed in sleep latency or sleep scores. These findings align with evidence that behavioural sleep interventions most consistently extend total sleep time [5,33,50]. Because sleep duration is not susceptible to learning or familiarisation effects, these changes likely represent a true intervention response. Given the established role of adequate sleep duration in supporting recovery and readiness in athletes [1,51,52,53], these improvements provide a strong foundation for interpreting subsequent physiological and performance outcomes.

Endocrine analyses revealed timepoint-specific differences in salivary cortisol between conditions, with no statistically significant differences at baseline. The basal cortisol response, which typically reflects a moderate surge within 30–45 min of waking [20], was lower (21.72%) following SHP than nSHP, though this difference did not reach statistical significance. Although improved sleep regularity and quality may influence nocturnal HPA-axis activation and circadian stability [54,55], normal diurnal cortisol variability provides an alternative explanation, and the present findings do not allow causal attribution of basal cortisol differences to these mechanisms. In contrast, a balanced morning cortisol response supports efficient glucocorticoid signalling, which may help limit sympathetic overactivation before exercise and conserve energy for performance tasks [22]. The observed cortisol secretion patterns, particularly at timepoints with statistically significant between-condition differences, including pre-anaerobic and post-aerobic testing, are consistent with stress-related regulatory mechanisms, while percentage differences at non-significant timepoints should be interpreted descriptively rather than inferentially. The lower post-exercise cortisol concentrations observed under SHP at aerobic testing timepoints suggest an attenuated HPA-axis response to aerobic load rather than dysregulated variability [56]. Such modulation may reflect improved arousal regulation and a reduced physiological stress burden during exercise, although these inferences remain speculative. Nonetheless, this pattern is consistent with evidence that sleep loss elevates evening cortisol, delays its quiescent phase, and disrupts neuroendocrine control even after short exposure [21,25,56,57]. Short-term sleep loss also reduces vigilance and alters autonomic balance [56], mechanisms that may plausibly contribute to the greater hormonal volatility observed under nSHP [58].

Although salivary cortisol responses were clearly altered after the sleep hygiene protocol, mediation analyses did not support cortisol as a significant mediator between sleep hygiene and performance outcomes. Importantly, these analyses were exploratory and not designed to establish causal mechanisms. The mediation analyses identified statistically significant total and direct effects for vertical jump height and peak repeated sprint ability, while all corresponding average causal mediation effects (ACMEs) were non-significant, indicating no evidence that cortisol mediated these performance outcomes. This pattern suggests that performance improvements associated with sleep hygiene are likely driven by non-endocrine mechanisms, including neurobehavioral and neuromuscular pathways, with minimal contribution from the cortisol response [52,53,59]. This interpretation accords with evidence that sleep optimisation, particularly sleep extension or strategic naps, improves explosive and high-intensity sport-specific outputs and cognitive contributors to performance, whereas endocrine markers, including cortisol, do not consistently explain variability in short-duration or high-power performance tasks [49,60,61,62,63]. Observational and mechanistic work on athletes shows that irregular sleep–wake timing, activating pre-sleep behaviours, and suboptimal sleep hygiene are associated with poorer sleep indices, which were explicitly targeted by the components of the SHP [50]. Consensus guidance reinforces sleep regularity and adequate sleep opportunity as determinants of health and performance, supporting the behavioural foundation of the protocol implemented [64]. In basketball and related jumping tasks, better sleep indices are associated with more favourable force–time characteristics, consistent with the observed improvements in VJ outcomes; conversely, sleep-loss models report decrements in vigilance and autonomic regulation that degrade explosive and sprint performance independent of changes in cortisol [8,60,62,63,65]. The absence of statistically significant mediation aligns with evidence that sleep interventions can improve performance via direct neurobehavioural and neuromuscular pathways (e.g., psychomotor vigilance, motor unit recruitment, movement efficiency) without requiring endocrine mediation. Reviews consistently show that sleep loss impairs sport-specific execution and cognitive contributors to performance, resulting in reduced explosive and sprint outputs independent of endocrine changes [8,66].

Adherence to sleep hygiene components varied over the intervention period, suggesting that behavioural regulation may have been influenced by environmental and competitive demands. Athletes initially adhered to several principles, with gradual declines and moderate compliance over time. Although adherence patterns were examined descriptively across the intervention period, formal quantitative analyses of adherence variability (e.g., variance or time-trend modelling) were not performed; therefore, interpretations of adherence fluctuations should be considered contextual rather than inferential. These patterns may reflect behavioural fatigue, circadian disruption, or limited control over sleep environments, as reported in athletic populations [53]. These findings align with previous observations that sustaining consistent sleep behaviours is challenging for athletes exposed to irregular training and travel schedules [24,53]. Variability in adherence likely reflects differences in the feasibility and perceived utility of specific behaviours in real-world contexts, reinforcing the need for individualised sleep hygiene interventions that account for contextual constraints and behavioural fatigue [67,68]. In contrast, inconsistent adherence has been associated with sleep fragmentation and impaired recovery processes [69]. Reinforcing adherence during high-demand periods may help sustain sleep quality and recovery capacity, as suggested by prior research [5,23,53].

Several limitations should be acknowledged when interpreting these findings. The final sample size was modest, and exclusions after enrolment reduced statistical power, particularly for mediation and exploratory analyses. Adherence and sleep behaviours were assessed using self-reported measures, which may be subject to recall and reporting bias, and objective verification of adherence was not available. Participants were drawn from a single collegiate soccer team, limiting the generalisability of the findings to other athletic populations, competitive levels, or sporting contexts. The sleep hygiene intervention was multi-component and individualised, which restricts attribution of effects to specific behaviours. Cortisol sampling was limited to discrete timepoints and did not permit assessment of full diurnal rhythms or the cortisol awakening response, nor formal testing of variability. Finally, although a crossover design reduced inter-individual variability, the study was not powered to detect small indirect effects, and mediation analyses should therefore be interpreted cautiously. The sequencing of performance tests may have introduced fatigue or carry-over effects; however, this risk was mitigated by administering the repeated sprint ability test before the Yo-Yo IR1 test and including a 24 h recovery interval, which was intended to preserve neuromuscular integrity before aerobic assessment. Although evidence suggests that sleep hygiene effects are largely reversible and behaviour-dependent [24,53,68], educational carry-over cannot be fully excluded in a crossover design. Participants may have retained awareness of sleep hygiene principles during the subsequent nSHP condition, and formal statistical tests of carry-over or order effects were not performed. This limitation should be considered when interpreting condition-specific effects.

The findings of the current study have practical implications for collegiate-level soccer player preparation and recovery, particularly regarding sleep behaviour. Implementing structured sleep hygiene (SHP) as a low-cost behavioural strategy within daily training routines may support sleep duration and selected performance-relevant outcomes. Core principles, such as maintaining regular bed- and wake-times, minimising evening light exposure and pre-sleep stimulation, and optimising sleep-conducive environments, represent modifiable behaviours with established benefits for sleep duration and continuity. For athletes who use stimulants, both caffeine dose and timing are critical; randomised controlled evidence shows that consuming approximately 400 mg of caffeine within 12 h of bedtime delays sleep onset, alters sleep architecture, and increases sleep fragmentation, underscoring the need for individualised caffeine cut-offs around training and competition demands [23,53]. When schedules permit, sleep extension or strategic napping may complement SHP, as supported by prior work [23,49,53]. Triangulation of endocrine, performance, and mediation data supports an integrated interpretation of the findings, although future studies should assess other plausible mediators, such as heart-rate variability, inflammatory cytokines, or anabolic hormones, to further elucidate mechanisms. Future studies should incorporate these physiological markers alongside objective sleep assessments (e.g., actigraphy or polysomnography) to refine mechanistic understanding and test potential interactive effects. Larger and more diverse samples are also needed to confirm generalisability and to determine whether SHP effects persist under varying competitive and environmental conditions.

In summary, the structured SHP improved sleep duration, was associated with timepoint-specific modulation of cortisol responses, and coincided with select improvements in explosive and sprint performance, with no evidence of cortisol-mediated effects. While other aerobic and anaerobic indices showed non-significant or emerging trends, these outcomes align with the broader literature emphasising sleep regularity and sufficiency as foundational to athletic performance and health. By promoting consistent sleep-related behavioural regulation, SHP emerges as a practical and modifiable component of athlete preparation. Embedding individualised SHP education within athlete monitoring and recovery frameworks could enhance adherence and facilitate the translation of sleep-related improvements into sustained performance and recovery gains.

## 5. Conclusions

This study demonstrates that an acute, structured SHP was associated with measurable changes in sleep behaviour, selected performance outcomes, and timepoint-specific endocrine responses. Implementation of the SHP was associated with significant improvements in selected performance metrics, including vertical jump height and peak sprint ability, while mediation analyses indicated that these effects occurred independently of cortisol. Endocrine findings showed timepoint-specific reductions in cortisol concentrations, most notably in the anticipatory and post-exercise phases, without evidence of sustained baseline or diurnal regulation. These findings suggest that sleep hygiene may influence stress-related endocrine responses during exercise-relevant periods, while performance improvements appear to arise through non-endocrine pathways, such as behavioural or neuromuscular mechanisms. Collectively, the results position sleep hygiene as a practical, low-cost, although temporary, behavioural intervention that can support sleep duration and selected performance-relevant outcomes in female collegiate soccer players. Assuming that active reinforcement, monitoring, or contextual support for proper sleep hygiene is provided, our findings reinforce its relevance within evidence-based training and recovery strategies.

## Figures and Tables

**Table 1:** Key Components of the Sleep Hygiene Protocol.

Component	Recommendation
1.	Avoid all electronic devices (TV, phones, computers) after 20:00
2.	If electronic devices are used, cease at least 30 min before bedtime
3.	Use blue light filters or “cool” light settings from 19:00
4.	Wear blue-light blocking glasses before bed
5.	Dim ambient lighting after 21:00
6.	Use low-wattage bulbs in the bedroom
7.	Maintain a constant bedroom temperature of 19–20 °C
8.	Avoid caffeine and supplement consumption after 17:00
9.	Consume the last main meal 2–3 h before bed
10.	Drink warm milk before sleeping
11.	Drink warm chamomile tea before sleeping
12.	Take a warm shower or bath before bed
13.	Wear eye masks whilst sleeping
14.	Wear earplugs whilst sleeping
15.	Remove time indicators (e.g., clocks) from the room
16.	Avoid mid-sleep disruptions (e.g., bathroom use)
17.	Target a minimum of 8 h of sleep per night
18.	Take a short nap (<30 min), and refrain from napping after 14:00

**Table 2:** Cortisol Responses (nmol/L) Across Testing Conditions Before and After Physical Tests.

Timepoint Comparison	nSHP Mean ± SD (nmol/L)	SHP Mean ± SD (nmol/L)	Δ % Change	*p*	ES_g_
Baseline	8.93 ± 8.16	6.99 ± 7.22	−21.72%	0.600	0.20
Pre-Anaerobic (RAST)	5.25 ± 3.57	2.99 ± 1.55	−43.05%	0.006	0.84
Post-Anaerobic (RAST)	3.19 ± 1.94	2.74 ± 1.92	−14.10%	0.150	0.38
Pre-Aerobic (YYIR-1)	4.08 ± 2.78	3.06 ± 1.87	−25.00%	0.060	0.55
Post-Aerobic (YYIR-1)	9.86 ± 6.72	4.36 ± 3.10	−55.78%	0.004	0.90
Pre vs. Post-Anaerobic (nSHP)	5.25 → 3.19		−39.23%	0.01	0.68
Pre vs. Post-Aerobic (nSHP)	4.08 → 9.86		+58.62%	0.035	0.57
Pre vs. Post-Anaerobic (SHP)	2.99 → 2.74		−8.36%	0.225	0.28
Pre vs. Post-Aerobic (SHP)	3.06 → 4.36		+29.81%	0.340	0.23

Note: Δ = Change from post to pre; ES_g_ = Hedge’s g effect size; RAST = Repeated Anaerobic Sprint Test; SHP—Sleep Hygiene Protocol; nSHP—No Sleep Hygiene Protocol; YYIR-1 = Yo-Yo Intermittent Recovery Test Level 1; SD = Standard Deviation.

## Data Availability

The data presented in this study are available from the corresponding author upon reasonable request. The data are not publicly available due to institutional ownership by the affiliated tertiary institution.

## Abbreviations

The following abbreviations are used in this manuscript:

SHP	Sleep Hygiene Protocol
nSHP	Non-Sleep Hygiene Protocol
AUC	Area Under the Curve
VJT	Vertical Jump Test
YYIR-1	Yo-Yo Intermittent Recovery Test-1
RAST	Repeated Anaerobic Sprint Test
